# Observations on a Peculiar Species of Convulsions in Children

**Published:** 1825-04

**Authors:** John North

**Affiliations:** Surgeon.


**Art. II.**—***Observations on a peculiar Species of Convulsions in Children.*** By John North, Surgeon.

In the observations which were offered by me in the January Number of this Journal, upon a peculiar convulsive affection of children, I had one, and only one, object in view: to state what I believe to be the fact, and to oppose opinions which I conceive to be founded in error. In the course of the communication to which I allude,* I referred particularly to the case of Mr. Prall's child, which I stated to be affected with the "crouping noise," although it was otherwise in good health. Since January, this child has suffered much from painful dentition, and from paroxysms of convulsive breathing. Relief was always experienced immediately by a free division of the gums. The bowels have not been costive, but the motions were of an unhealthy appearance. No symptom of determination of blood to the head has been observed. The thumbs were almost constantly and rigidly bent towards the palm of the hand, but a relaxation was observed after lancing the gums. In the morning of the 17th of February, I was requested instantly to see the child. Not a moment was lost; but it was dead before my arrival at the house, although not more than ten minutes had elapsed from the time of the attack which I am about to describe.

The child was considered by its parents, who were always anxiously watching it, to be in better health than usual. It was very lively, and for the last day or two the bowels had been regular. Without being inclined to dwell upon trifles, I shall state the manner in which the child was attacked. It was lying in the nurse's lap, playing with the bell-rope, cheerful and apparently well. Its amusement was interrupted by the arrangement of some part of its dress: a very violent and passionate fit of crying was induced; a paroxysm of the crouping noise came on, with convulsive breathing; a livid hue was observed around the mouth; and the child died.

Whether this fact, which I have felt myself bound to record, after the observations which I had previously made upon the case of this child, will be thought to militate against, or even entirely to subvert, the opinions I have endeavoured to maintain, I do not stop to determine. My only object in discussing this question, is to arrive at the truth, and I publish it as freely as if it afforded my previous statement full and undeniable support. To be mistaken is very pardonable; but to present to the profession only one-half of a case, for the purpose of gaining converts to a favourite opinion, is a moral enormity of no ordinary magnitude. The sudden death of this infant, however, does not alter my opinion. I have never ventured to state that the train of convulsive symptoms, under which this child laboured, is independent of the brain: I have only objected, and I do still object, to the unqualified assertion that such a state will certainly be followed by *hydrocephalus*, unless we have recourse to a particular mode of treatment, which is constituted by repeated bleeding, and *three* grains of calomel every *three*

* *London Med. and Phys. Journal,* January 1825, p. 41.

hours. Such was the practice that was recommended by very high authority, in the case of another child of Mr. Prall's, who, during the period of dentition, laboured under *all* the symptoms that I have formerly described. It was not, however, had reccourse to. A year and a half have elapsed, and the child affords a living instance that it was not necessary.

If, during this convulsive state, there should be evidence of determination of blood to the head,—if the head should be hotter than natural, the countenance flushed, together with febrile disturbance in the system, it would follow, as a matter of course, that our attention would be directed particularly to the head, and that our treatment would be modified accordingly. But I still think that the mere occurrence of the crouping noise, even if in connexion with the contracted thumb, does not justify the practitioner in instantly determining that effusion upon the brain will succeed, and that the most energetic practice is instantly to be enforced.

I have no observations to add, with respect to the treatment I consider necessary, to those I submitted in my former communication.

I consider this child of Mr. Prall's to have died from a sudden and overwhelming rush of blood to the head, differing but little from an attack of sanguineous apoplexy in an adult, and rendered probable by the violent emotion excited at the moment, in the manner I have above described. They who are inclined to support a contrary opinion may, perhaps, contend that the sudden death of the child was an occurrence to be expected from the previous symptoms. To this I should reply, that the child had been for a fortnight in apparently good health; and hence we could not infer that any lasting impression had been made upon its constitution, which would be productive of sudden death; and, secondly, that all the symptoms had more than once vanished by freely lancing the gums, which would not have been the case if they had arisen from any serious cerebral affection. I would ask, if it should unfortunately happen that Mr. Prall's son, who has now for a year and a half been in comparative good health, should suddenly be cut off in a fit, would it be justifiable to presume that his death was the result of some particular affection of the head, which we had reason to expect would occur, although the symptoms which are said to characterise that state had disappeared for so long a time?

Some interesting cases, of a similar nature, have lately been published by Mr. C.* I perfectly agree with that gentleman in the view he takes of the disease, and in the treatment he adopts. Its origin may be various. It may depend entirely upon pain-

* *London Medical Repository*, February.

ful dentition,—upon deranged abdominal secretions,—and may be accompanied by, or perfectly free from, any symptoms denoting cerebral affection. Our plan of treatment will, of course, be modified as the disease may assume different characters.

The appearances which were observed upon examination post mortem, which was made about thirty-six hours after death, I shall now briefly describe. My friend, Mr. Stone, was kind enough to assist me. I particularly wished him to be present at the examination, as I was aware that we were rather at issue as to the original character of such diseases.—The vascular system of the brain presented a highly turgid appearance. A small quantity of fluid was effused under the dura mater, in several parts. I doubt whether the ventricles contained more than the usual quantity of fluid which is found in them. The whole cerebral mass was particularly firm. The cerebellum was softer than it usually is at so short a period after death. It was not thought necessary to examine the thorax. The only evidence of disease we detected in the abdomen, was seated in the liver: its whole substance was very firm, and upon its upper surface were three white spots, each about the size of a sixpence, which felt, upon passing the scalpel over them, like cartilage. There was no appearance of disease in the mesentery or intestines.

*Seymour-place, Bryanstone-square; February.*

---

### Art. III.—*Case of Perforation of the Stomach.*

By J. R. Griffiths, Esq.

